# Influence of hydraulic clam dredging and seasonal environmental changes on macro-benthic communities in the Southern Adriatic (Central Mediterranean Sea)

**DOI:** 10.1186/s12862-023-02197-9

**Published:** 2024-01-04

**Authors:** Roberto Carlucci, Giulia Cipriano, Daniela Cascione, Maurizio Ingrosso, Enrico Barbone, Nicola Ungaro, Pasquale Ricci

**Affiliations:** 1 Department of Biosciences, Biotechnologies and Environment (DBBA), University of Bari, Bari, Italy; 2 CoNISMa, P.le Flaminio, 9, 00196 Rome, Italy; 3 Interdepartmental Research Center for Coastal Dynamics, University of Bari, Bari, Italy; 4 Department of Civil, Environmental, Land, Building Engineering and Chemistry (DICATECh), Polytechnic University of Bari, Bari, Italy; 5 Environmental Prevention and Protection Agency of Puglia Region, Scientific Direction U.O.C. Natural Environment - Regional Sea Centre, Bari, Italy

**Keywords:** Benthic assemblages, *Chamelea gallina*, Indicator of Value (IndVal), Species diversity, Feeding traits, Shellfish harvesting

## Abstract

Macro-benthic communities on the shallowest soft bottoms are impacted by hydraulic dredgers used for the harvesting of the striped venus clam (*Chamelea gallina*). Changes in macro-benthic assemblages were analysed across four areas in the Southern Adriatic Sea (Central Mediterranean Sea) during the winter and summer 2022 (low and high fishing pressures, respectively). Two sampling surveys were carried out collecting abundance data (N/100m^2^) of 69 species. Diversity indices (Shannon–Weiner and Equitability) were calculated and differences between seasons and areas were tested using non-parametric tests. Changes in the assemblage and feeding habits were explored by adopting permutational multivariate analysis of variance on 34 species. Moreover, environmental conditions of all areas in the two seasons were characterized by a set of 8 variables. Seasonal changes in diversity were only detected for the Shannon–Weiner index, with values significantly higher in winter than summer. Macro-benthic assemblages differed between the two seasons, and the winter assemblages were well-distinct in each area. In contrast, a high overlap was shown in the summer assemblages of the four areas. Changes in feeding habits showed an increase in filter feeding polychaetes and opportunistic/scavenger species during the summer. Temperature, Salinity and primary production were positively correlated to summer stations indicating potential thermic stress on the assemblages. The results provide information on the benthic community impacted by dredge disturbance and seasonal changes driven by environmental conditions, stressing the need to integrate these aspects in assessments on the health status of marine ecosystems required by European directives and fishery management plans.

**Supplementary Information:**

The online version contains supplementary material available at 10.1186/s12862-023-02197-9.

## Introduction

Benthic communities distributed on shallow soft bottoms are subject to natural and anthropogenic stress drivers, leading to spatio-temporal changes in the structure of assemblages and diversity because of their synergic or antagonistic interactions [1]. The ability to assess the effect of multiple disturbances on such kinds of communities, and to identify the crucial impacting drivers, remains a difficult challenge. Indeed, the effects on the community of several anthropogenic impacts (e.g., fishery, nutrient and organic matter enrichment, anchoring, extraction of non-renewable resources, etc.) can mask changes induced by natural environmental disturbances, and vice versa [2]. This is particularly true for the soft bottoms exploited by dredge and trawling gears [3]. In particular, the harvesting of commercial shellfishes with mechanical fishing gears, such as hydraulic dredges operating on the shallowest bottoms, exerts direct and indirect negative impacts on the benthic communities [4]. In particular, the impact of hydraulic dredgers on the bottoms is characterized by mechanical disturbance on and into the seabed surface, with physical damage to the organisms [5, 6], and resuspension of the sediment in the water column with increased turbidity of the water [7]. However, these benthic communities are generally distributed in highly dynamic environments, with several natural stresses (e.g., water column variables, sediment composition, water circulation, river run flows, etc.) playing a fundamental role on their structure [8, 9]. Several studies have been conducted to understand these ecological dynamics in the case of clam dredge impacts, reporting a high complexity of this interactive condition and the difficulty to assess the recovery of communities after disturbance [10–12]. Therefore, the information derived from these studies is indispensable for the management of resources according to an Ecosystem-based Management approach (EBM) [13].

Benthic communities are a fundamental component of marine ecosystems and the assessment of their health status to support conservation and management actions as required by the EU Water Framework Directive (WFD) 2000/60/EC [14] and Marine Strategy Framework (MSFD) Directive 2008/56/EC [15, 16]. In particular, for the MSFD, the assessment of Descriptor 6 (Integrity of Seafloor) can be achieved through the analysis of the pressure intensity on the benthic habitat, as well as the response of communities to fishing through their spatio-temporal changes [17]. At present, the effective implementation of MSFD measures suffers from knowledge gaps in understanding ecological interactions, tipping points, and thresholds [18]. Therefore, the need to acquire knowledge on these aspects becomes a fundamental milestone, especially in large areas historically exploited by dredge fishing.

In the Mediterranean Sea, the shallowest soft bottoms host important benthic communities exploited by hydraulic dredgers targeting the striped venus clam (*Chamelea Gallina*, Linnaeus 1758), an infaunal filter-feeder bivalve that inhabits the Well Sorted Fine Sands (WSFS) biocenosis [19]. The fishing areas distributed along the Italian Adriatic coasts are the most important in terms of clam production [20]. In particular, in the southern Adriatic along the northern Apulian coast, the fishing grounds cover an area of over 300 km^2^ between 1 to 10 m in depth [21]. The fishing effort distribution is divided between the fleets harbouring in Barletta and Margherita di Savoia and those of the North Gargano area [22]. Although significant in terms of production, information on the effects due to hydraulic dredgers on the substrate and macro-benthic assemblages are still little investigated in the area. One attempt dates back to the late 1990s [23, 24], whilst the most recent information was collected through the implementation of a monitoring plan inherent to the stock assessment of *C. gallina* in 2013 [22]. Partial information on the benthic communities in areas with high and low fishing impacts have been acquired from an experimental survey in the small area of the North Gargano [25]. No less relevant, knowledge on seasonal changes in these benthic communities from winter to summer have not been investigated, nor have the relationships with the main environmental variables.

The goal of this study was to explore the seasonal and spatial differences of benthic assemblages occurring within the fishing grounds impacted by hydraulic dredgers in the Southern Adriatic Sea. The assemblages were investigated in terms of changes in structure and diversity, by identifying characteristic taxa and functional groups in winter and summer seasons. Different fishing pressure and environmental conditions were detected in these two sampling periods.

## Material and methods

### Study area

The dredge fleet exploiting the clam beds along the Apulian coasts in the Southern Adriatic Sea are organized in two Management Consortia (Barletta and North Gargano), which administered the corresponding geographical areas (or fishing compartments), according to the Italian regulations [26]. These areas are characterized by several geomorphological traits, which contribute to the occurrence of the well-sorted fine sands (WSFS) biocenosis [27], and the development of striped venus clam beds. The dynamics of this biocenosis are exposed to different environmental conditions between the northern Gargano area and that of Barletta-Margherita di Savoia (hereafter Margherita) [28]. The former area is affected by the hydrographic dynamics of the northern Adriatic Sea and sediment inputs from the Po River [29], while the latter area is characterized by limited circulation and high sedimentation rate due to several river inputs distributed along the coast from Manfredonia to Barletta [30]. The North Gargano coastline is characterized by wetlands, as the Lesina and Varano lagoons in the western and middle zones, while raised beach deposits in proximity of calcareous rocky cliffs occur in the eastern zone [31]. The urbanization is very low and concentrated in few zones with touristic harbor or lagoon channels, as Rodi Garganico, Foce Varano and Lesina. On the other hand, Margherita and Barletta areas are characterized by a high degree of urbanization in the Barletta coast (over 90,000 inhabitants, with industrial ports and plants), and the presence of the largest marine saltworks in Italy along the Margherita coast, extended for about 20 km, and connected by channels with the sea [32]. In addition, several rivers are distributed in the northern area influencing the sediments dynamics and the spatial variation of benthic communities [30].

**Fig. 1:**
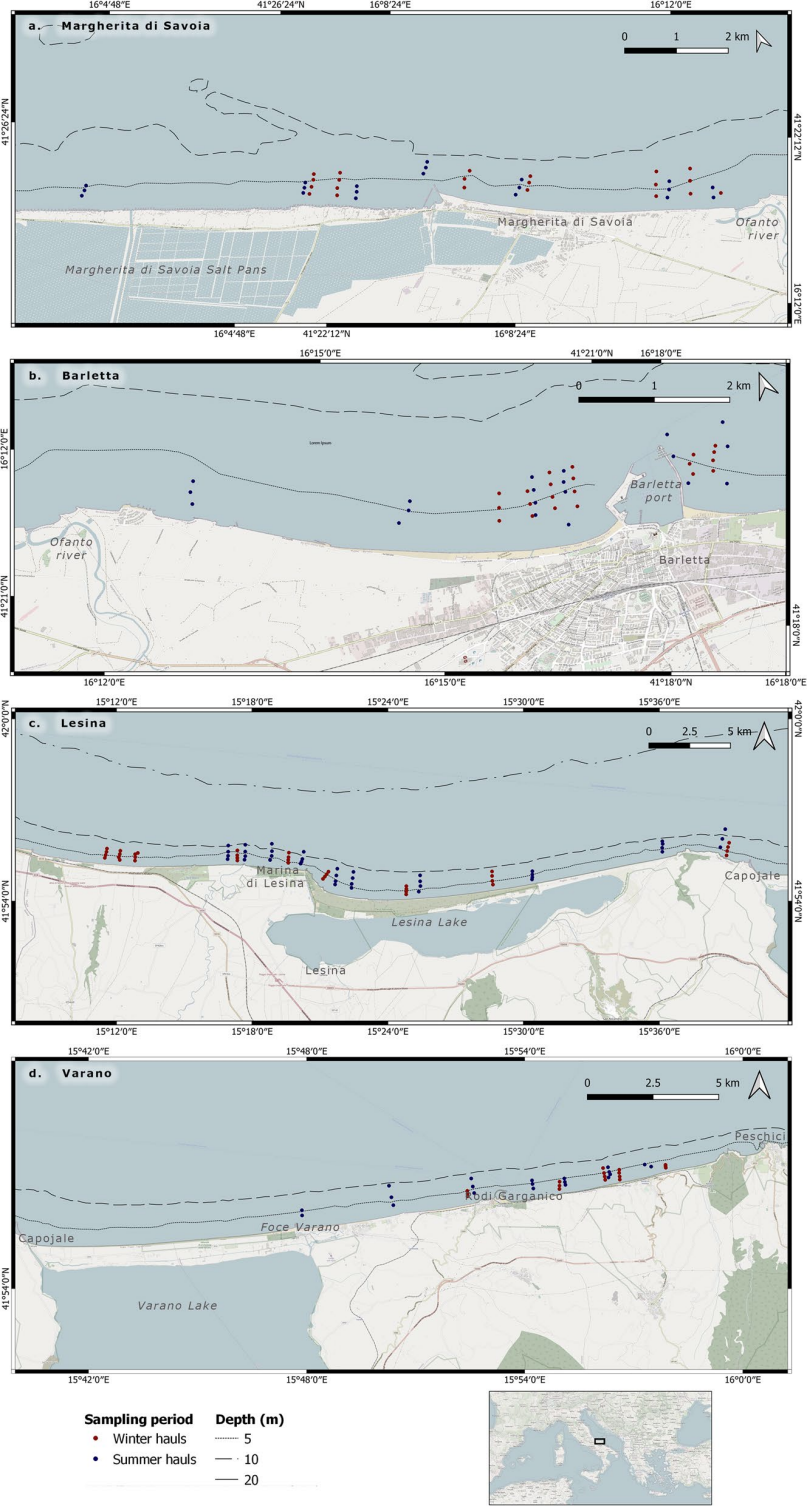
**a**-**d** Maps of study areas: **a** Margherita di Savoia, **b** Barletta, **c** Lesina, **d** Varano. Points indicate the sampling stations in the winter (red) and summer (blue) periods

Since the 1980s, these bottoms are exploited for the harvesting of clams and other commercial shellfishes [24, 33, 34], sustaining about 6% of the fishery production in the area [35]. To date, within each fishing compartment, two areas are exploited by four fishing fleets (Fig. 1a-d). The Barletta and Margherita fleets (12 vessels for each fleet) operate in the zones located to the south and north of the mouth of the Ofanto River, respectively (Fig. 1a, b). The Barletta fishing areas are smaller than those of Margherita, and fishermen tend to be self-regulating in their fishing activities, with 4 out of 12 dredges used each month to harvest clams, to maintain a high price on the fishing market, as well as to preserve the clam stock (personal communication). Differently, the larger extension of the fishing grounds in the Margherita area induces the entire fleet to exploit the clam beds, working all dredges in the same period. The North Gargano compartment covers the area between Peschici and the mouth of the Saccione River (Fig. 1c, d). Here, the fleets are based in the Lesina and Capoiale harbours, with the former exploiting fishing grounds from Punta Pietre Nere up to the western zone. The latter operate on the bottoms between Punta Pietre Nere and Peschici. Overall, the dredges fleet of this compartment counts a total of 44 vessels, often all working together [22].

### Survey periods and sampling procedures

Experimental surveys were carried out during winter (since December 2021 to February 2022) and summer seasons (July 2022) (Table 1). To identify the status (no-fishing or fishing) and intensity of fishing pressure within each area and period, information on the number of operative dredges for each fleet and fishing days were collected. This collection was carried out involving the fishermen, fishing cooperatives and Management Consortia, and successively, the information were validated by comparing national and European references data, such as those of official stock assessments of the Scientific, Technical and Economic Committee for Fisheries (STECF), when available. Information was acquired from official fishermen's logbooks, landing declarations of Consortia, and informal reports. Notably the reconstruction of the fishing status was a critical issue due to administrative changes in the composition of Management Consortia, especially in the North Gargano area, often requiring informal contacts to define the effective effort in terms of fishing days and vessels employed in the fishing activities.

**Table 1:**
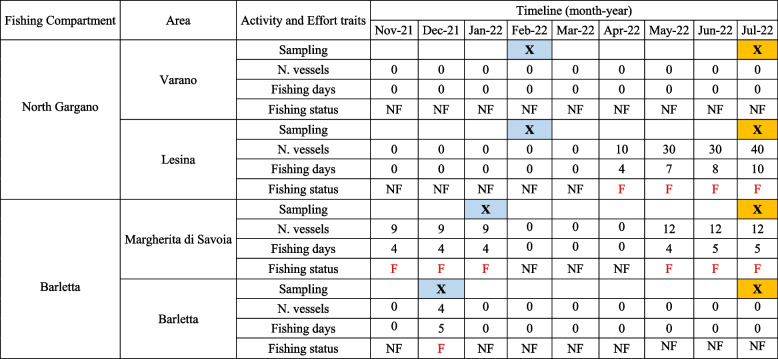
Summary of sampling periods (winter in blue, and summer in orange) and fishing effort traits (number of vessels and fishing days) collected from fishermen and logbooks reports in each investigated area of the two fishing compartments. The monthly fishing status (no-fishing, NF and fishing, F) within each area is reported

Overall, fishing activities were absent, or medium–low in some areas, during the winter, while they were more frequent and intense in summer (Table 1). Due to unfavourable meteorological conditions from October 2021 to December 2021 there was no fishing activity in the North Gargano compartment. In addition, the eastern fishing area over the Rodi Gargano harbour has not been exploited since 2013, with only occasional fishing days [22]. In the Barletta fishing compartment, a very low fishing pressure was detected for the Barletta area in December 2021, while a medium pressure occurred in the Margherita di Savoia fishing grounds located in proximity of the Ofanto river mouth since November 2021 to January 2022. The Barletta fleet exploits very small zones located around the harbour focused on the eastern area. Overall, this condition of both fishing compartments is confirmed by the landing data of 2021, reported in the official clam stock assessment of the STECF [36], in the which no landings are reported for the northern Gargano compartment, while a total landing of 213 tonnes is reported for the Barletta one.

During the period May–July 2022, the Lesina and Margherita areas experienced an increase in the fishing pressure, while in Barletta and Varano areas, fishing activities remained absent during these months. In particular, the highest intensity of fishing pressure was observed in the Lesina area, with approximately 30–40 dredges active 7 to 10 days per month (Table 1). In the Margherita area, the fishing pressures was characterized by the activity of the entire fleet (12 dredges) employed for 4–5 days per month.

The information on the summer fishing status for the North Gargano area, acquired by the fishermen and fishing cooperatives involved in monitoring survey, represented mean monthly values of fishing days reconstructed through meeting and interviews, while the number of dredges was confirmed by official licences registered to the Management Consortium. Differently, fishing effort data obtained for the Barletta compartment are acquired by direct requests to the Management Consortium. It should be noted that, for the year 2022, no official STECF clam stock assessment reports are yet available. However, the temporal snapshot of the state of fishing in each area can be considered highly realistic given the integration of different official and informal data sources.

Data on macro-benthic assemblages were collected during surveys addressed to assess the status of the striped Venus clam*,* according to the sampling protocol defined in the framework of the “Dredgers Molluscs Survey “ research program [26]. In each investigated area, the sampling procedure was carried out using commercial fishing vessels operating in the fishing compartments. Linear transects were located perpendicular to the coastline spaced about 2 km from each other in a depth range of 2–10 m, according to fishermen’s indications on the main fishing grounds exploited in the previous years. Along each transect, the sampling was carried out using the fishing dredge up to a maximum of four sampling stations spaced approximately 0.25 nautical miles apart, when the *C. gallina* (as a commercial resource) predominates in the catch. Otherwise, the sampling operation was moved to the following transect. In each station, the dredger was towed parallel to the coastline. The operators on board collected information about the operation time, depth (m), geographical position and the haul length by means of a Geographical Positioning System (GPS, Garmin 650 model). A total of 58 and 56 stations were sampled in winter and summer seasons, respectively (Fig. 1).

The dredge used in the sampling procedure was characterized by a 3 m wide mouth. A schematic illustration of the dredge is shown in Fig. S1. Within the dredge, a net sampler with 14 mm mesh, 40 cm wide and 20 cm long was fixed in the mouth, to harvest *C. gallina* juveniles and associated epifauna [21]. The catch in each haul was weighed on board and it was divided into sub-samples when it exceeded 2 kg. Samples obtained by this procedure were stored at –20 °C to be sorted later and classified at the lowest taxonomic level when it was possible. Individuals of each species or taxon were counted, and the wet weight was measured to a precision of 0.1 g.

### Data collection

Data were standardized for all sampled species using the area swept by dredge in each sampling station, calculated from data of the haul length multiplied by the width of dredge. Thus, the standardized abundance of each species (N 100 m^−2^) was organized in a taxa-stations matrix, excluding the target species of the monitoring (*C. gallina)* to avoid bias in the analysis due to its very high abundance.

The environmental conditions in the water column were described for each station using variables obtained from the Copernicus database (http://marine.copernicus.eu/), according to the sampling periods. This choice was due to lack of environmental data directly collected in situ. Biogeochemical variables were derived from the physical reanalysis component of the Mediterranean Monitoring and Forecasting Centre. The environmental variables were estimated in a spatial grid of square cells (length of cell side = 4 km), where the values were detected along bathymetric strata. In our analysis, the environment variables acquired from Copernicus were bottom temperature (T bot, °C), Salinity (Sal, PSU), dissolved oxygen (Diss. O_2_, mmol m^−3^), pH, dissolved phosphates (PO_4_, mmol m^−3^), dissolved ammonium (NH_4_, mmol m^−3^), dissolved nitrates (NO_3_, mmol m^−3^), and the net primary production (NPP, mg m^−3^ day^−1^). Within each cell, the mean value of a variable was calculated from the values of the available depth strata, and from data collected during the week of the sampling date (weekly mean). The weekly mean value was assigned to the sampling stations falling within the corresponding cell and values are reported in the Table S1. The procedure was carried out using a multiband regular raster projected in the Coordinate Reference System (CRS) WGS84 using the R package *raster* (version 3.5–2) [37] in the R Studio environment (version 1.3.1093) working with R language (version R-3.6.3) [38].

### Data analysis

Seasonal and spatial differences in species diversity and in the structure of the benthic assemblages were analysed. Before the analysis, the *taxa* not identified at a minimum of family level, and those belonging to the faunal category of fishes (non-target species of dredge), were excluded (Table S2). For the diversity indices (Shannon–Weiner, H’ and Equitability or Pielou, J) the median values were calculated (min–max, and I and III quartiles), and differences between seasons and compartments were tested using the non-parametric Kruskall-Wallis (KW) test; the *post-hoc* comparison was carried out by means of the Mann–Whitney (U) test with Bonferroni’s correction [39]. Statistical tests were carried out using PAleontological STatistics software (PAST ver. 4.10) [40].

Differences in the structure of the benthic assemblage were explored using multivariate analysis. In this step, taxa characterized by a frequency occurrence lower than 5% on all hauls were excluded (Table S2), and abundance data were transformed by fourth root, in order to maintain a balance between rare and very abundant species [41]. A similarity matrix was calculated using the Bray–Curtis (BC) similarity index in order to run the multivariate analysis.

The seasonal and spatial differences in the assemblage were tested using a multifactorial model based on three fixed orthogonal factors (Season, 2 levels Winter and Summer; Depth, 2 levels < 4 m, ≥ 4 m; Area, 4 levels Margherita di Savoia, Barletta, Lesina and Varano). Null hypotheses, assumed as no temporal and spatial differences in the multivariate location and dispersion, were tested using permutational multivariate analysis of variance (PERMANOVA) [42], and the permutational analysis of multivariate dispersions (PERMDISP) [43]. In PERMANOVA, the permutation method adopted was “Permutation of residuals under a reduced model”, calculating *p-values* through 9999 permutations [44]. Single factors, or interactions between pairs of fixed factors, with a significant *p-value* (*p* < *0.05*) were analysed using the *post-hoc* PAIRWISE t-test to evaluate differences within their levels calculating the Monte Carlo *p-values*. The PERMDISP test was conducted with 9999 permutations to calculate the differences between the mean distance of centroids for the most important interactions between factors.

Temporal and spatial patterns of assemblage were visualized using an unconstrained ordination method, namely Principal COordinate analysis (PCO) [45]. The relative species contribution to the ordination of sampling stations was assessed using the Pearson’s correlation coefficient (ρ).

The multivariate analysis was also performed by aggregating taxa into Functional Groups (FGs) obtained from the information on both their faunal category and feeding habit, according to the classification proposed by Bremner [46]. The categories “Filter feeder” (Ff), “Deposit feeder” (Df), “Opportunistic-Scavenger” (OS), and “Predator” (P) were adopted to describe the feeding habits, which were acquired from the global database [47], and the literature [5, 48]. A total of 14 FGs were obtained by the combination of faunal category and feeding habits (Table S2). The data matrix “FGs × stations” was analysed performing the same procedures adopted for the taxa-hauls matrix. In addition, the distance between centroids for the levels of the “Season × F. area” interaction was calculated and visualized using the PCoA plot. Multivariate analyses were conducted using PRIMER 6 + PERMANOVA software [44, 49].

To identify the characteristic species during the two seasons with different fishing pressures, the Indicator of Value (IndVal) [50, 51] was calculated on the overall macrobenthic assemblage (aggregation of all fishing areas), and within each fishing area. The IndVal is expressed by the following equations:

$$IndVal = A \times B$$

where, the indicator combines the specificity of a species (A, its relative abundance in a group of observations), with the species fidelity (B, relative frequency of occurrence of the species within a given group of observations). Species with an IndVal greater than or equal to 0.30 were considered the characteristic species of the assemblage, and the IndVal values of winter and summer were compared. The calculation of IndVal was carried out using the labdsv R package (version 2.0–1) [52].

The environmental conditions in each area were described by medians (min–max, I and III quartiles) of environmental variables values previously acquired. Statistical tests on environmental variables were carried out using the non-parametric KW test, and *post-hoc* comparison was carried out through the U test with Bonferroni’s correction. In addition, the correlation between environmental variables was calculated using the Pearson correlation coefficient (ρ). Finally, environmental variables were plotted in the PCO ordination as vectors, to better visualize their contribution to the organization of the benthic assemblage structure.

## Results

**Fig. 2:**
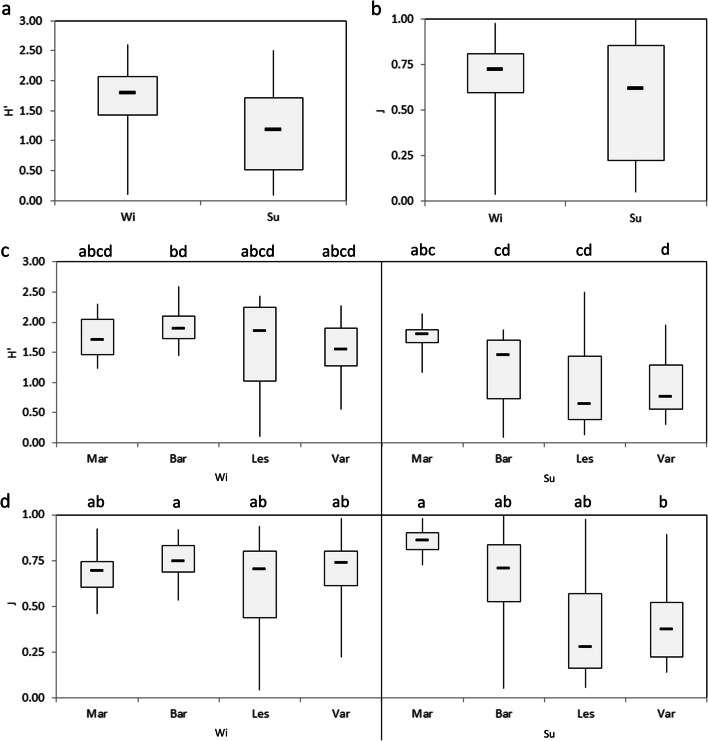
**a**-**d** Boxplots of diversity indices (Shannon, H’ and Pielou, J) calculated between (**a**, **b**) the two seasons (Winter, Wi; Summer, Su), and (**c**, **d**) across the fishing areas (Margherita di Savoia = Mar; Barletta = Bar; Lesina = Les, and Varano = Var). Different letters beside the box plots indicate significant differences in diversity indices between areas across seasons based on the Mann–Whitney pairwise Bonferroni-corrected test (U, *p* < 0.05)

A total of 69 taxa were collected and identified in both sampling periods: 14 decapod crustaceans, 6 echinoderms, 36 molluscs (21 bivalves, 13 gastropods, 1 scaphopod and 1 cephalopod), 1 nemertine, 10 polychaetes, 1 sipunculid, and 1 stomatopod, (Table S2). Overall, 26 were collected in both seasons, 34 and 9 taxa were exclusively sampled in the winter and in the summer, respectively. A significant reduction in diversity was exclusively observed between the seasons for the Shannon index (Fig. 2a, b; Table S3). The median H’ value in winter (1.80, Interquartile Range IR = 0.64) was significantly higher than that in summer (1.19, IR = 1.21; χ^2^ = 19.62; *p* < *0*.*001*).

During the winter, the H’ index was ranged between the highest median values in Barletta (1.88, IR = 0.33) and Lesina (1.85, IR = 1.22) and the lowest in the Varano area (1.54, IR = 0.62) (Fig. 2c). During the summer, a reduction in the H’ median values was observed for all zones, except for Margherita di Savoia (1.89, IR = 0.19). The lowest median values were detected for the Lesina and Varano areas (0.64, IR = 1.05; 0.76, IR = 0.74, respectively). Overall, the median value of Barletta was significantly higher than those of Barletta, Lesina and Varano in summer (*p* < *0.01*, Table S2). In addition, the median value in Margherita was significantly higher than that of Varano during the summer (U = 12, *p* < 0.05). Considering the Equitability index, significant differences were detected only between Varano in summer (0.37, IR = 0.30) and those of Barletta in winter (0.74, IR = 0.15) and Margherita in summer (0.86, IR = 0.09) (*p* < *0.05*, Table S2, Fig. 2d).

### Structure of benthic assemblages

**Fig. 3:**
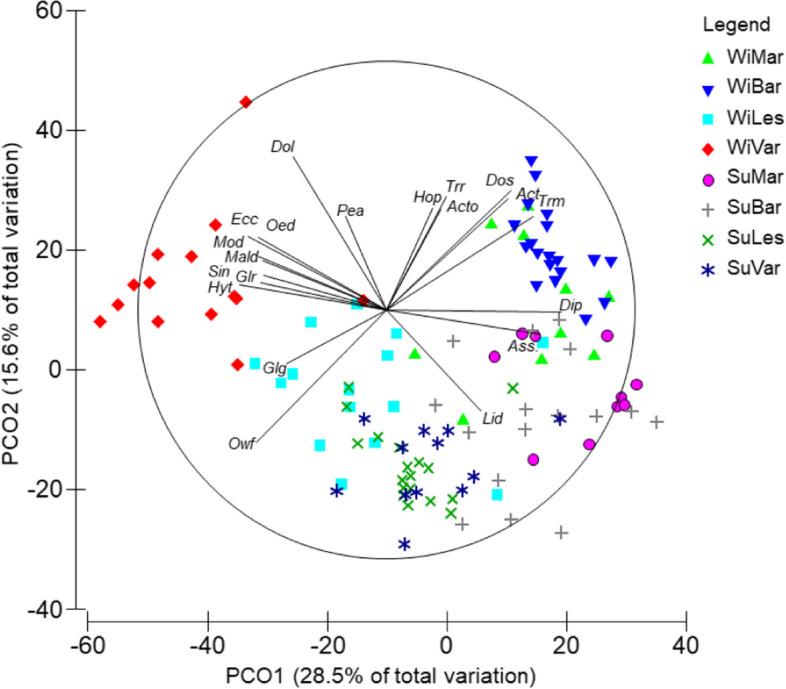
PCO ordination plot of sampling stations based on the Bray–Curtis similarity calculated by taxa. Stations are labelled as “Season × Area” interaction levels (WiMar, Winter Margherita di Savoia; WiBar, Winter Barletta; WiLes, Winter Lesina; WiVar, Winter Varano; SuMar, Summer Margherita; SuBar, Summer Barletta; SuLes, Summer Lesina; SuVar, Summer Varano). Vectors indicate the correlation of FGs to the multidimensional space according to Pearson’s correlation coefficient (*ρ* > 0.4). Taxa are coded as: *Acanthocardia tuberculata* (Act), *Acteon tornatilis* (Acte), *Astropecten spp.* (Ass), *Diogenes pugilator* (Dip), *Dosinia lupinus* (Dol), *Donax semistriatus* (Dos), *Echinocardium cordatum* (Ecc), *Glycimeris glycimeris* (Glg), *Glycera rouxii* (Glr), *Hyalinoecia tubicola* (Hyt), *Holothuria poli* (Hop), *Liocarcinus depurator* (Lid), Maldanidae (Mald), *Moerella donacina* (Mod), *Oestergrenia digitata* (Oed), *Owenia fusiformis* (Owf), *Peronidia albicans* (Pea), *Sipunculus nudus* (Sin), *Tritia mutabilis* (Trm) *Tritia reticulata* (Trr)

**Fig. 4:**
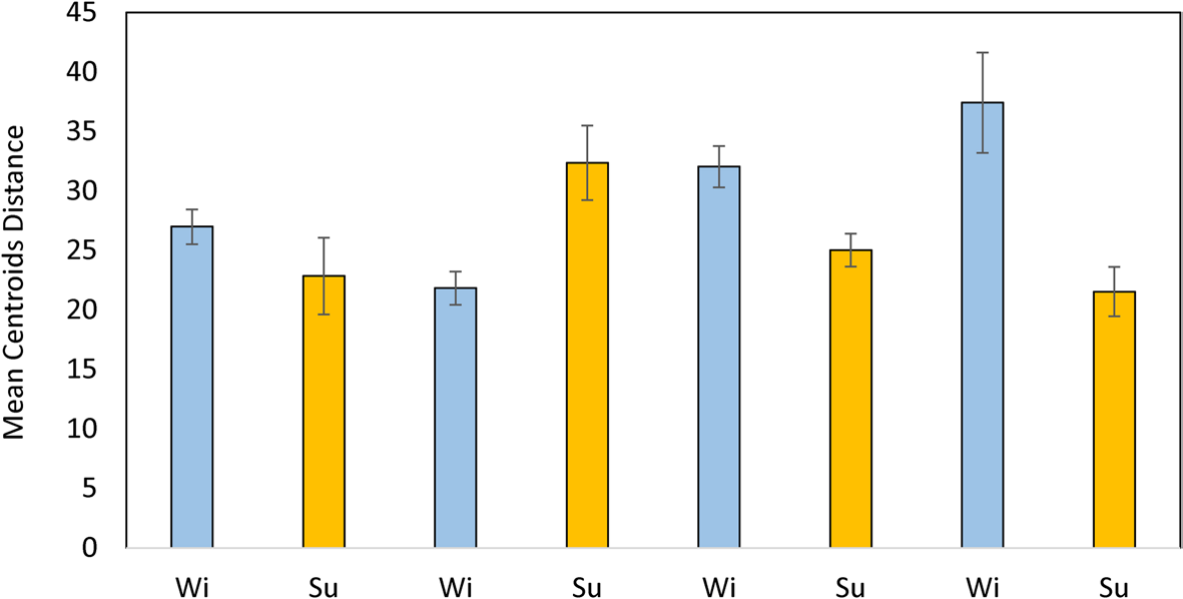
Mean centroid distance (± SE) obtained by the PERMDISP test carried out on levels of the “Season × Area” interaction. Wi and Su indicate Winter and Summer, respectively

**Fig. 5:**
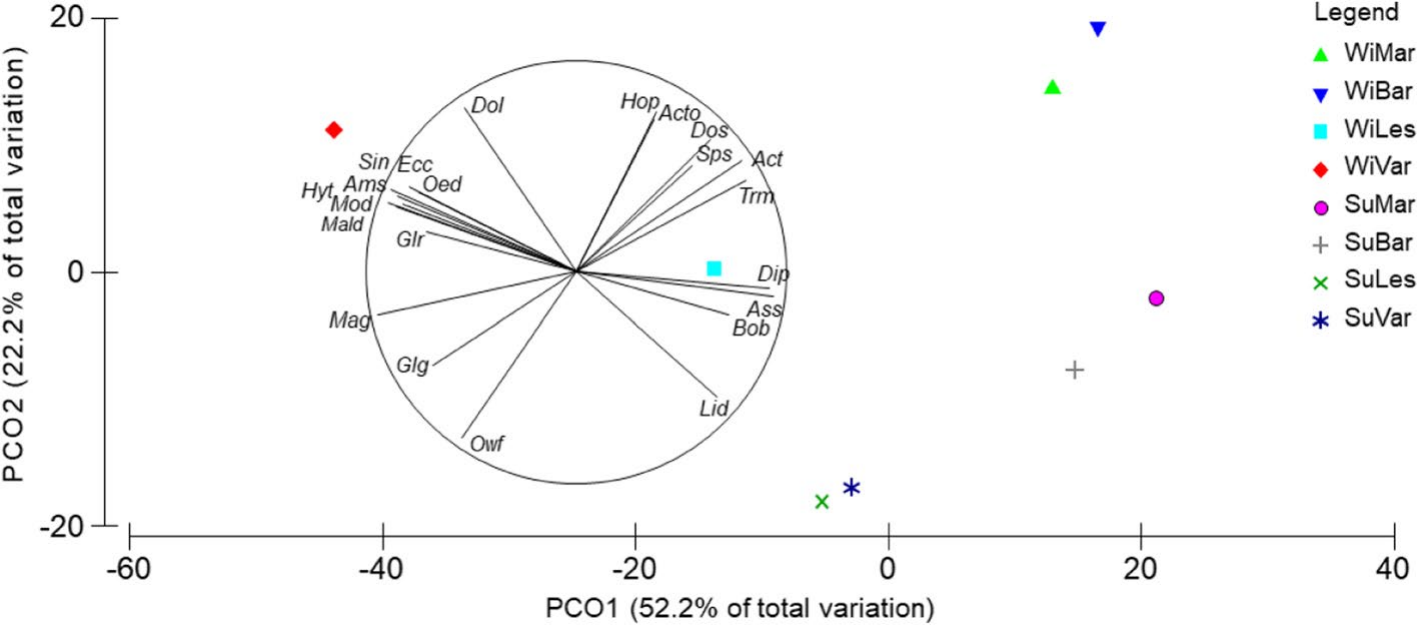
PCO ordination plot of the distance between centroids calculated by taxa and aggregating the stations within the levels of the “Season × Area” interaction (WiMar, Winter Margherita di Savoia; WiBar, Winter Barletta; WiLes, Winter Lesina; WiVar, Winter Varano; SuMar, Summer Margherita; SuBar, Summer Barletta; SuLes, Summer Lesina; SuVar, Summer Varano). Vectors indicate the correlation of taxa to the multidimensional space according to Pearson’s correlation coefficient (*ρ* > 0.75). Taxa are coded as: *Acanthocardia tuberculata* (Act), *Acteon tornatilis* (Acto), *Amphiura spp*. (Ams), *Astropecten spp.* (Ass), *Bolinus brandaris* (Bob), *Diogenes pugilator* (Dip), *Dosinia lupinus* (Dol), *Donax semistriatus* (Dos), *Echinocardium cordatum* (Ecc), *Glycera rouxii* (Glr), *Glycimeris glycimeris* (Glg), *Hyalinoecia tubicola* (Hyt), *Holothuria poli* (Hop), *Liocarcinus depurator* (Lid), *Mactra glauca* (Mag), Maldanidae (Mald), *Moerella donacina* (Mod), *Oestergrenia digitata* (Oed), *Owenia fusiformis* (Owf), *Sipunculus nududs* (Sin), *Spisula subtruncata* (Sps), *Tritia mutabilis* (Trm)

PERMANOVA test showed significant differences between seasons, areas and the “Season × Area” interaction, while differences between depth layer were not statistically significant (Table 2, PERMANOVA test). The PCO ordination plot performed on the whole dataset showed a separation between winter stations along the first axis (28.5% of the total explained variation, Table S4), while the division between winter and summer stations occurred along the second axis (15.6% of the total explained variation, Fig. 3). The separation of stations in the winter period followed a geographical gradient, where Barletta and Margherita stations were plotted on the right part of the ordination, while Varano and Lesina stations were on the opposite side. The taxa which mainly correlated to the Barletta area were *Acanthocardia tubercolata, Acteon tornatilis, Holoturia poli, Donax semistriatus, Tritia reticulata* and *Tritia mutabilis* (*ρ* > 0.4). Moving towards the stations of Margherita, *Astropecten spp*. and *Diogenes pugilator* were the main correlated species. In Varano stations, the main taxa were *Dosinia lupinus, Peronidia albicans, Echinocardium cordatum, Oestergrenia. digitata, Moerella donacina, Glycera rouxii,* as well as polychates of Maldanidae, *Sipunculus nudus* and *Hyalinoecia. tubicola*. In addition, *Glycimeris glycimeris* marked a transition between the Varano and Lesina areas, with *Owenia fusiformis* as the taxa most correlated to Lesina winter stations. The summer stations were found to overlap greatly with the winter stations and their positions tended to blend with the winter stations of Lesina and Margherita. *L. depurator* was the main species correlated to the summer stations. This high overlap between the summer stations was confirmed by the PERMDISP results, which showed significant differences in the multivariate dispersion exclusively for the stations of Barletta with those of Lesina and Varano (Table S5). Moreover, the comparison of the multivariate dispersion in each fishing area between the two seasons showed significant changes in Barletta, Lesina and Varano (Fig. 4). The dispersion in the former area increased during the summer (t = 2.704; *p* < 0.05), while reductions in mean centroid distances were detected for the stations of Lesina (t = 3.037; *p* < 0.01) and Varano (t = 2.882; *p* < 0.05). The spatio-temporal pattern of stations was better visualized through the plotting of their centroids in the PCO (Fig. 5). Here, areas were well-distinguished along the PCO1 axis (52.2% of total explained variation, Table S4), with a large distance between their centroids, confirming the geographical separation of macro-benthic assemblages in both seasons. In winter, the Barletta and Margherita areas were more similar than those of Lesina and Varano. Along the PCO2 axis (22.2% of total explained variation), the separation of centroids reflected the seasonal differences in assemblages. In particular, the centroids of summer stations were closer to each other than those of the winter stations, with Lesina and Varano centroids highly overlapped. The difference in species composition observed between the Varano and Barletta areas during the winter was confirmed, with other species highly correlated (*ρ* > 0.75), such as *Amphiura spp*. and *Mactra glauca* in Varano, and *Spisula subtruncata* in Barletta. In the summer, *D. pugilator*, *Astropecten spp.* and *Bolinus brandaris* and *L. depurator* were the main taxa correlated to all areas, with the latter more associated at Lesina centroid. In addition, *O. fusiformis* showed a negative correlation to the PCO2 axis (*ρ* = -0.77), indicating the high occurrence of species in the Lesina area during the winter, and its increasing abundance during the summer in the entire North Gargano compartment.

**Table 2:** PERMANOVA results based on the Bray–Curtis similarity matrix of the benthic assemblages tested according to factors (Season Se, Area Ar, Depth De), and PAIRWISE t-test obtained for the Se × Ar interaction. The degrees of freedom (df), Sum of Squares (SS); Mean Sum of Squares (MS); Pseudo-F test; *p-value*s by permutations (P), and number of permutations (perms) are reported for each factor. In the PAIRWISE t-test, the t-test value (t), Monte Carlo *p-values* (P(MC)), and the average similarity (Av. S. %) are reported between Ar pairs (Margherita, Mar; Barletta, Bar, Lesina, Les; Varano, Var)

PERMANOVA results						
**Source of variation**	**df**	**SS**	**MS**	**Pseudo-F**	**P**	**perms**
Season (Se)	1	18960	18960	20.371	0.0001	9930
Depth layer (De)	1	1185	1184.6	1.2728	0.2523	9930
Area (Ar)	3	40911	13637	14.652	0.0001	9900
Se × De	1	1141	1140.6	1.2255	0.2937	9924
Se × Ar	3	14953	4984.4	5.3555	0.0001	9923
De × Ar	3	2935	978.26	1.0511	0.4023	9896
Se × De × Ar	3	2629	876.42	0.94166	0.5582	9878
Res	98	91211	930.72			
Total	113	188210				
PAIRWISE test						
Se × Ar interaction—Winter level						
**Ar pairs**	**t**	**P(MC)**	**perms**	**Av. S. (%)**		
Mar, Bar	2.444	0.0001	9947	56.8		
Mar, Les	2.575	0.0002	9947	46.0		
Mar, Var	3.774	0.0001	9950	22.4		
Bar, Les	4.674	0.0001	9938	38.7		
Bar, Var	5.145	0.0001	9942	22.6		
Les, Var	2.997	0.0001	9939	31.8		
Se × Ar interaction—Summer level						
**Ar pairs**	**t**	**P(MC)**	**perms**	**Av. S. (%)**		
Mar, Bar	1.111	0.2917	9948	57.1		
Mar, Les	3.495	0.0001	9932	49.2		
Mar, Var	2.643	0.0002	9959	54.0		
Bar, Les	2.527	0.0001	9933	50.0		
Bar, Var	2.012	0.0036	9945	51.7		
Les, Var	2.319	0.0004	9920	60.3

**Fig. 6:**
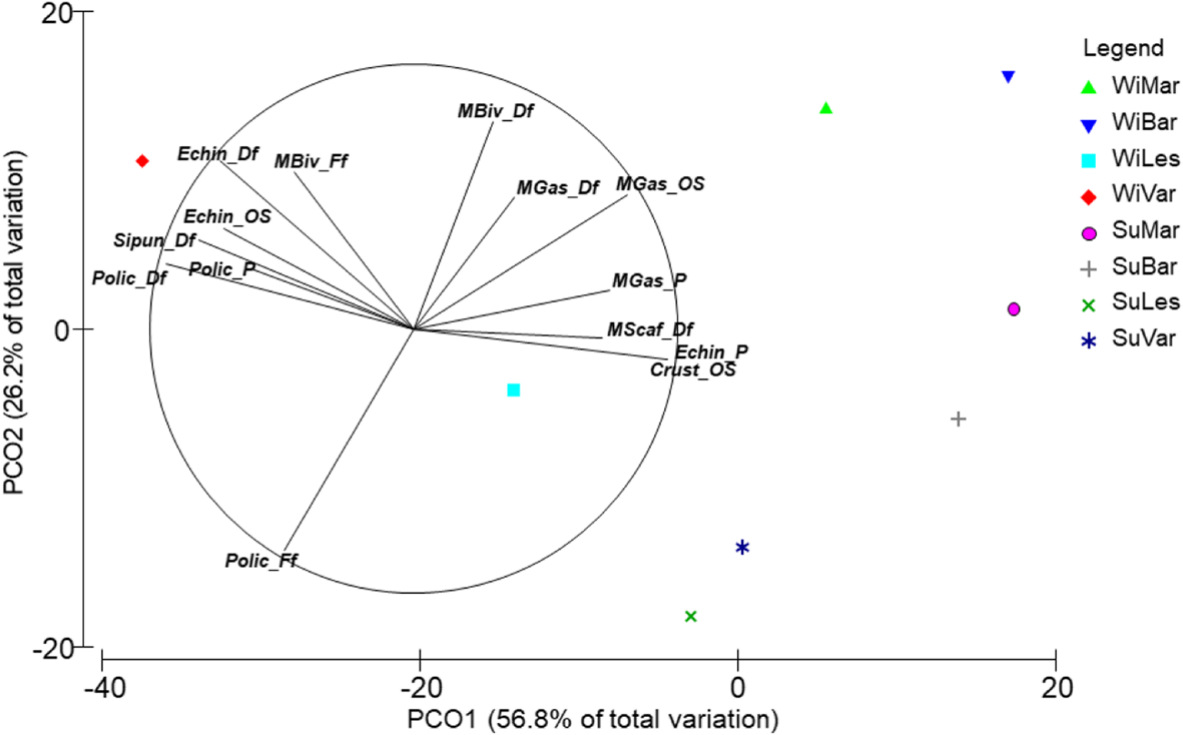
PCO ordination plot of the distance between centroids calculated by functional groups aggregating the stations within the levels of the “Season × Area” interaction. (WiMar, Winter Margherita di Savoia; WiBar, Winter Barletta; WiLes, Winter Lesina; WiVar, Winter Varano; SuMar, Summer Margherita; SuBar, Summer Barletta; SuLes, Summer Lesina; SuVar, Summer Varano). Vectors indicate the correlation of FGs to the multidimensional space according to Pearson’s correlation coefficient (*ρ* > 0.4). FGs are coded in Table S2

The analysis of FGs confirmed the spatial and temporal differences in benthic assemblages observed in the previous analysis (Fig. 6). The distance between centroids calculated within the “Season × Area” interaction levels showed a large separation of winter benthic assemblages along the PCO1 axis (56.8% of total explained variation, Fig. 5; Table S4). In particular, Varano was characterized by the FGs of deposit feeders belonging to echinoderms, polychaetes and sipunculids, bivalve filter feeders, polychaetes predators and opportunistic echinoderms. In contrast, the Margherita and Barletta areas showed high correlations of bivalve deposit feeders and opportunistic/scavenger gastropods and deposit feeders. Along the second axis (26.2% of total explained variation), the separation between the areas followed the seasonal periods. The Barletta and Margherita assemblages showed an increase in the abundance of opportunistic/scavenger crustaceans, echinoderms and gastropod predators, and scaphopod deposit feeders. On the other hand, changes detected for the Lesina and Varano areas were represented by the increasing of polychaetes filter feeders, with a high negative correlation (*ρ* = -0.70 with the PCO2 axis), being the most important groups associated to both areas in the summer. Overall, changes from absent/low fishing pressure (winter) to a high fishing pressure (summer) seems to be characterized by the sharp decrease in abundance of deposit feeders and the increase in the abundance of polychaetes filter feeders in the North Gargano area, and opportunistic/scavenger species on Barletta and Margherita bottoms.

**Fig. 7:**
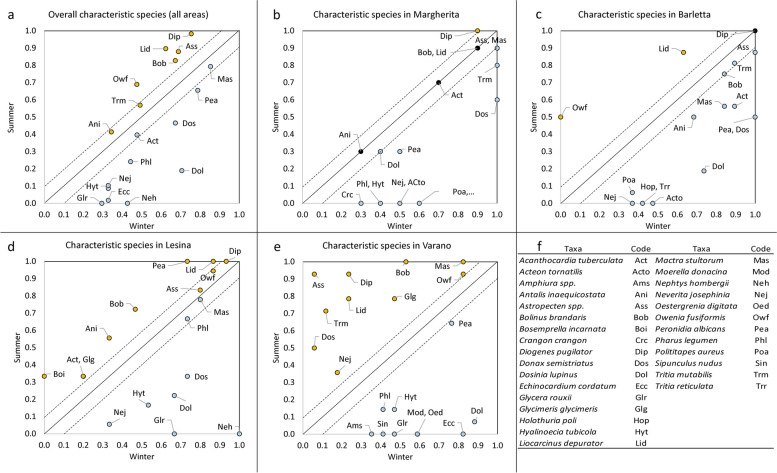
**a**-**f** Comparison between Indval values between winter and summer (on the x and y axes of the plots, respectively) in both overall and single areas. Those taxa showing relatively equal values are black-coloured; species with a higher IndVal value in one of the two seasons are either blue (winter) or orange-coloured (summer)

The Indval analysis on the characteristic species confirmed the general pattern of seasonal changes in the benthic assemblages driven by the fishing impact and environmental variations (Fig. 7a-e). In the winter, the most characteristic species of all the areas investigated were the bivalves, such as *Mactra stultorum*, *P. albicans*, *D. semistriatus*, *D. lupinus*, *Pharus legumen* and *A. tubercolata*, and some polychaetes (*Nephtys hombergii*, *G. rouxii* and *H. tubicola*) were found exclusively in this season (Fig. 7a). In the summer, the highest Indval values were observed for *D. pugliator*, *B. brandaris*, *Astropecten spp.*, *L. depurator* and *O. fusiformis*. Overall, Margherita area did not show relevant changes in IndVal values between the two seasons, with *D. pugilator* as the only species with a summer Indval value slightly higher than its winter one (Fig. 7b). Other characteristic species (*B. brandaris*, *Astropecten spp.*, *L. depurator*, *A. tubercolata*, and *A. inequicostata*) showed similar values in both periods. Differences in the seasonal assemblages were due to the exclusive/predominant occurrence of the bivalves *D. semistriatus* and *Polititapes aures*, the sea cucumber *H. poli,* the polychaete *H. tubicola*, the gastropod *Neverita. josephinia*, and the decapod *Crangon crangon*. Similarly, Barletta showed only two species (*L. depurator*, *O. fusiformis*) with higher IndVal values in summer than the winter, while *D. pugilator*, *Astropecten spp.* and *B. brandaris* showed similar values in both periods (Fig. 7c). The differences were due to the bivalves *P. aures* and *D. lupinus*, as well as the gastropods *T. reticulata*, *A. tornatilis*, *N. josephinia*, and *H. poli*, which occurred exclusively in the winter. In the North Gargano area, species characterized by summer IndVal values higher than those of the winter were very abundant (Fig. 7d, e). In particular, species of the Varano area showed the most relevant increase in IndVal values, while this proved more limited in Lesina. In the former area, *Astropecten spp.*, *D. pugilator.*, *L. depurator* and *T. mutabilis* showed summer IndVal values (> 0.70) much higher than those of winter (< 0.25). Notably, in this area, *D. pugilator* showed the lowest summer IndVal value compared to other areas. Other relevant characteristic summer species were *O. fusiformis*, *B. brandaris*, and *M. stultorum* with IndVal values higher than 0.90. The result of the latter species was interesting since it showed the highest IndVal values in all areas during the winter, except for that of Varano. *G. glycimeris* also proved to be a characteristic species in the Varano area, with the highest IndVal value reached in the summer (0.79). In addition, among the characteristic species of the winter assemblage in Varano, *E. cordatum* reached the highest Indval value (0.82) compared to other areas. Finally, in Lesina, the characteristic species of the summer were *L. depurator*, *P. albicans* and *B. brandaris*, while *D. pugilator*, *O. fusiformis* and *Astropecten spp.* had similar values in both seasons. Further differences were due to the higher values of *N. hombergii*, *G. rouxii*, *D. semistriatus* and *D, lupinus* (> 0.65) in winter than during the summer (< 0.35).

### Environmental traits of season and areas

**Fig. 8:**
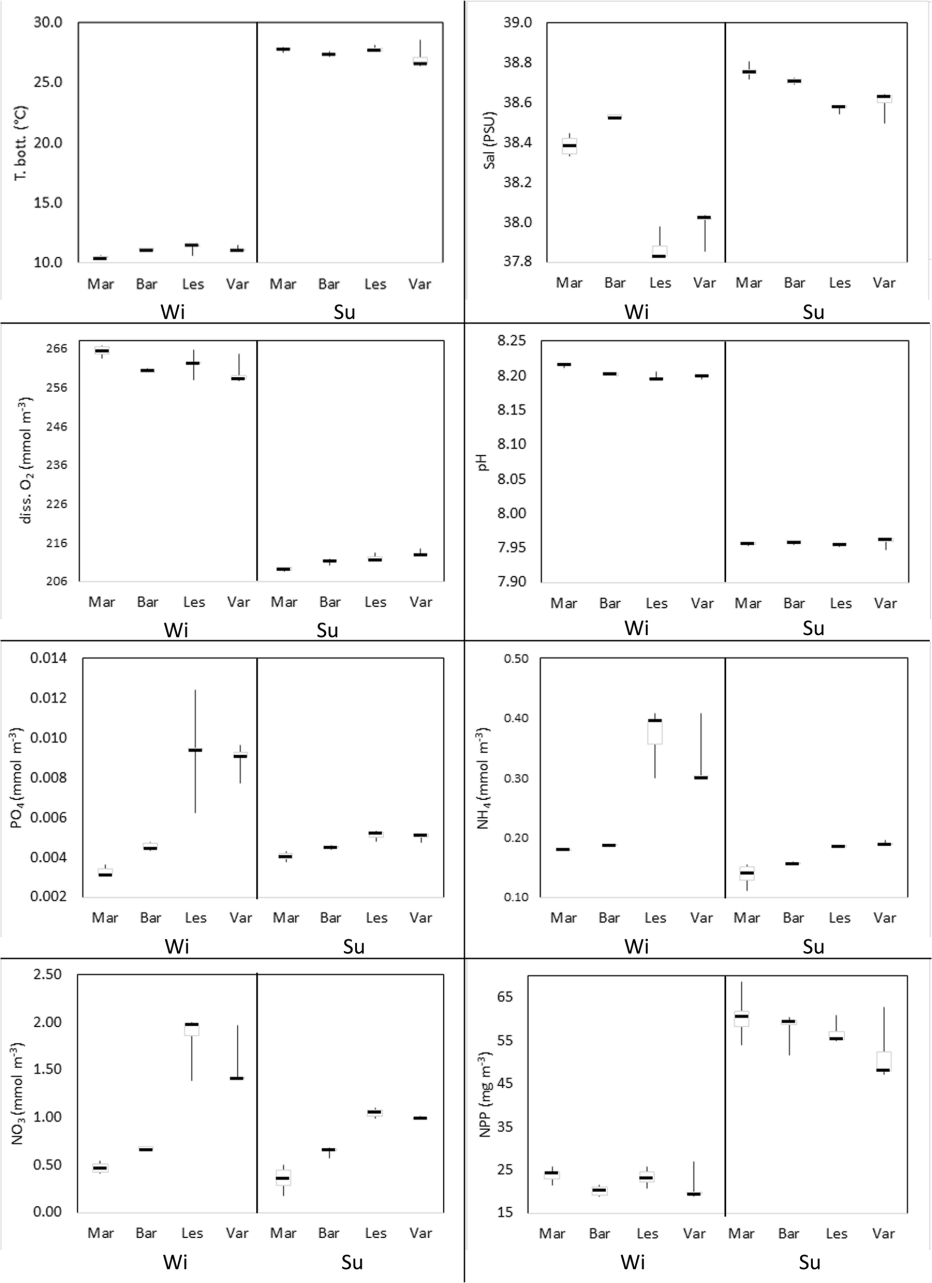
Boxplots of environmental variables analysed in both seasons within the investigated areas (Margherita di Savoia = Mar; Barletta = Bar; Lesina = Les and Varano = Var)

The Temperature, Salinity and Oxygen concentration in the water showed significant differences between all areas in the winter (Table S6, Fig. 8). The temperature showed the lowest median value in Margherita (10.4 °C) and the highest in Lesina (11.5 °C), while the highest median values of salinity were detected in Margherita and Barletta (38.4 and 38.5 PSU, respectively) and the lowest one in Lesina (37.8 PSU). In addition, the oxygen concentration showed very high median values in Margherita (265 mmol m^−3^) and the lowest in Varano (258 mmol m^−3^). In the same season, significant differences were found for pH, PO_4_, NH_4_ and NO_3_ among all investigated areas, with the exception of the Lesina and Varano stations. In these two areas, the concentration of nutrients was always higher than those detected in Barletta and Margherita. Finally, the net primary production (NPP) showed significant differences between the Margherita-Lesina and Barletta-Varano pairs (Table S6, Fig. 7). Specifically, the former pair showed the highest median values (24.4 and 23.4 mg m^−3^ day^−1^, respectively), while the lowest ones were in the latter pair (20.5 and 19.9 mg m^−3^ day^−1^, respectively).

In the summer, the differences between the areas were less evident (Table S6). Only the oxygen concentration, NH_4_ and NO_3_ showed statistically significant differences in all areas (Table S5). The former, showed the median values of Margherita (209 mmol m^−3^) and Barletta (211 mmol m^−3^) lower than those in Lesina (212 mmol m^−3^) and Varano (213 mmol m^−3^). Salinity showed an increase in median values compared to the winter, with values higher than 38.5 PSU. The PO_4_ concentration was significantly different in all areas, apart for Lesina and Varano (both median values of 0.005 mmol m^−3^). Notably, Lesina and Varano showed a high decrease in the value compared to the winter period (median values of 0.009 mmol m^−3^). Considering the temperature, Margherita and Lesina showed the highest median values equal to 27.8 and 27.7 °C, respectively, and significantly different from that of Barletta (27.4 °C, *p* < 0.001). Differently, Varano showed the lowest median value of temperature (26.6 °C), but it was not significant due to the widest range of values between 26.5 and 28.6 °C. Finally, the NPP did not show significant differences between the areas in the summer, but a relevant difference was detected between the median value in Varano (48.2 mg m^−3^ day^−1^) and those of other areas, which were higher than 55 mg m^−3^ day^−1^.

**Fig. 9:**
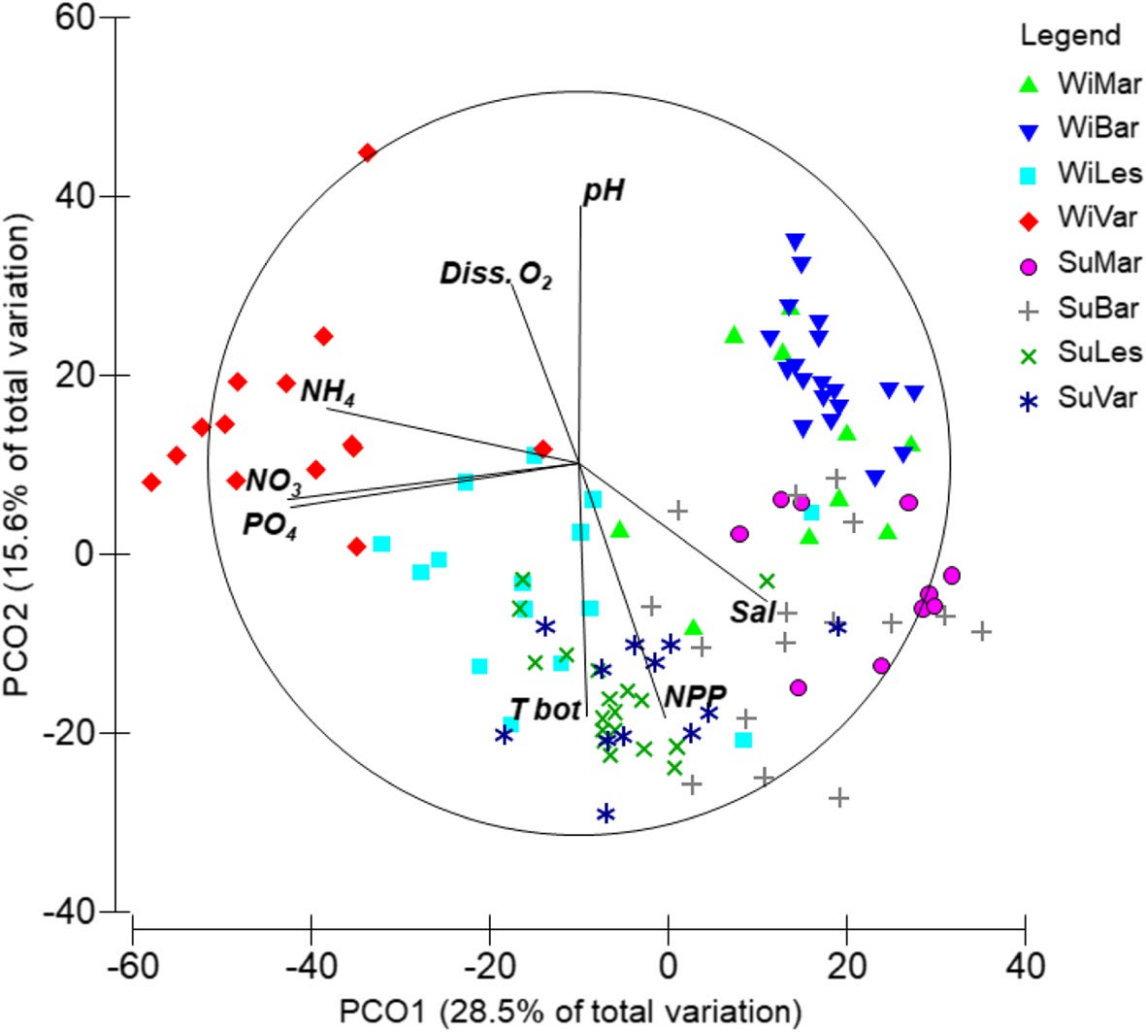
Environmental variables plotted as vectors in the PCO ordination based on the Bray–Curtis similarity calculated by taxa-stations matrix. Vectors are calculated according to the Spearman’s correlation coefficient (rs > 0.5) (for details on correlations between variables see Table S7). Environmental variables are coded as: bottom temperature (T bot), Salinity (Sal), dissolved oxygen (Diss. O2), pH, dissolved phosphates (PO4), dissolved ammonium (NH4), dissolved nitrates (NO3), and net primary production (NPP)

Correlations between variables are reported in Fig. 9 and Table S7. Temperature showed a positive correlation with the salinity and the net primary production (*p* < 0.001), while a negative correlation was detected with dissolved oxygen and pH (*p* < 0.001). Salinity was negatively correlated to the nutrient concentrations and positively to NPP (*p* < 0.001). Nutrient concentrations were positively correlated with each other and negatively to the NPP (*p* < 0.01). Considering the correlation between the variables and the station ordination in the PCO plot, Salinity was positively correlated to the PCO1 axis with stations in the Barletta and Margherita areas (Fig. 9). On the other hand, nutrients were negatively correlated to the first axis with high values in the Varano stations. Temperature and NPP were negatively correlated to the PCO2 axis characterizing all summer stations. Oxygen and pH were positively correlated to the second axis, indicating a strong reduction in their values during the summer. In addition, summer stations in Margherita and Barletta were more influenced by salinity values higher than those in the North Gargano area.

## Discussion

The study of the effects of dredge fishing pressure on the shallow benthic communities is a critical issue, mainly because of the species composition and their relative abundance can be affected by the natural stress associated to highly dynamic environments [2]. Thus, the changes driven by spatial variation in the sediments type and seasonal environmental factors, such as temperature, salinity, the oxygen, and energy available in the environment (as primary production), can mask the alteration in the structure of communities due to the mechanical impacts of the dredge on the bottoms. No less relevant, the species distributed in shallow benthic habitats are constantly influenced for their entire life cycles by several environmental disturbances, which include physical stress due to extreme events (e.g. storms) [4, 11]. As a consequence of this condition, the benthic species are adapted to a dynamic system tolerating several impacts, and the responses to stress are also driven by their life-history traits and interactions [3]. The complexity of the interaction between benthic communities and environmental factors make it difficult to fully understand the ecological characteristics of dredge-impacted areas from non-impacted ones [12]. However, the need to provide information for resource and ecosystem management has become urgent, especially in the context of climate change and in areas where knowledge about the impact of dredges is absent or very limited. To fill the gap on this aspect in large exploited marine ecosystems, it is important to assess the conditions of fishing grounds supporting a management of resources according to the EBM approach [13]. The results obtained in this study represent the first information on the seasonal changes (winter-summer) in the structure of benthic assemblages impacted by dredges operating in the Southern Adriatic Sea. These areas are the main fishing grounds in the Apulia region which are historically exploited for clam harvesting and affected by a decline in production [53], thus requiring attention in the management of these resources to ensure fishing productivity.

The first important evidence from our results is the geographical separation between the benthic assemblages sampled in the southern compartment of Barletta, and that of the North Gargano. This difference between the two compartments reflects their belonging to two contiguous, but different, biogeographic sectors in the Adriatic Sea (sectors 8 and 7, respectively) [54, 55]. In addition, the geographical separation was very marked during the winter, where each investigated area, in conditions of absence/low fishing pressure, show well-distinct macro-benthic assemblages. No differences between areas were detected in the summer, but a separation persisted at the level of compartments. Substantially, compartments are exposed to different environmental drivers, which contribute to the observed differences. In particular, the differences in benthic communities between the biogeographical sectors are mainly due to the features of sediments and coastal dynamics, which changes from the North Gargano to Margherita and Barletta coasts (Mastronuzzi e Sansò, 2002, [27, 31, 56]). Although the sediment granulometry is not considered in our study, since information in situ was not available, previous monitoring surveys on the status of commercial bivalves provide information on the biocenosis distribution in our study areas [22]. In particular, the WSFS biocenosis resulted more frequent and extended over 5 m of depth in the North Gargano bottoms. Differently, the main biocenosis distributed in the Barletta and Margherita bottoms were the superficial muddy sands in sheltered waters (SVMC) biocenosis, followed by those of coarse sands and WSFS. The pattern in the biocenoses spatial variation highlights the importance in the sediment sizes on the changes in the species composition between the two biogeographical sectors, in which the benthic communities have been developed and differentiated through several environmental stress and fishing impacts.

Starting from this condition, the geomorphological traits, the presence of freshwater river channels, coastal lagoons, and urban development levels in each investigated area draw a peculiar coastal landscape and water quality affecting the shallowest soft bottoms [27, 57, 58]. Indeed, the Barletta area is characterized by the influence of rivers discharges, as well as several anthropogenic pressures, such as a high level of urbanization along the coast and the industrial harbour [59]. On the contrary, the area of Margherita hosts the peculiar salt marches in the northern zone and limited urban development along the coastline [60]. The geographical position of the North Gargano coastline allows sediments coming from the northern Italian rivers to be retained and transported along the Italian coast [29]. The Lesina area is affected by several brackish and freshwater inputs along the coastline (e.g., Lesina lagoon channels and the Saccione and Fortore rivers). Therefore, this accumulation of sandy sediment provides favourable conditions for the establishment of the WSFS biocenosis throughout the North Gargano compartment up to 10 m in depth [22, 27]. Finally, differences between Lesina and Varano seem to be due to the rocky cliff behind the sandy beach extending from Rodi to Peschici [31]. In addition, this latter zone is affected by important flows of tourists during the summer, with the tourist port and beach resorts, with potential impacts on the coastal ecosystems [61].

The results show a seasonal change in the diversity of the assemblages detected only by the Shannon diversity index, where differences seem to be mainly due to the decrease in summer diversity median values in Varano and Lesina areas. A similar seasonal change has only been observed for mollusc assemblage impacted by dredges for the razor clam fishery in the Tyrrhenian Sea [12]. On the contrary, no significant differences were detected at macro-benthic community level in the Tyrrhenian Sea. A factor affecting this difference could be represented by different features of the two dredgers adopted in the harvesting. Indeed, the fishing gears operate at different towing speeds (higher for the striped Venus clam dredge), depths (shallower for the razor calm harvesting) and sediment layers, with a higher bottom penetration (7-10 cm) to catch razor clams. These features could explain the significant differences detected at the level of the overall macro-benthic assemblage in our study, where more species belonging to the epibenthic fauna and demersal domain are caught [62].

The winter period was useful to identify non-impacted areas (or those with very low impact) of the soft bottoms by the dredge pressure, with a separation of all investigated areas characterized by a specific assemblage. The Varano zone showed species linked to WSFS (*M. donacina*, *P. albicans*, *D. lupinus*) with the common heart urchin (*E. cordatum*)*,* a species with a fragile exoskeleton, considered highly vulnerable to the dredge impact [63]. This sea urchin could be affected by the excavation of the dredge on the bottoms, with an increase in its exposure and catchability, as observed in [11], where its occurrence is associated to intense fishing impacts in the Central Adriatic Sea. However, other environmental disturbances (e.g., strong storm events) could increase its exposure to harvesting, and our results seem to indicate the high abundance of *E. cordatum* in undredged bottoms, in line with observations reported in other studies [5, 12]. Indeed, negative winter weather conditions could transport sediment and organisms towards the shallowest bottoms. This observation also seems to be consistent with the high occurrence of *O. digitata*, which is a sea cucumber typical of muddy sand [64]. Its occurrence in sandy shallower bottoms, as observed in our results, could be due to storm events during the winter. Moving from Varano to the adjacent area of Lesina, the assemblage shift towards biocenosis characterized by the occurrence of the bivalve *G. glycimeris*, and the polycheates *N. hombergii*, *H. tubicola* and *O. fusiformis*. This change in species composition seems to indicate a passage towards environments with a higher presence of sediment resuspension and muddy sand, likely to be due to the presence of channels and river mouths.

In the Barletta compartment, differences between assemblages were less marked, but the effect of the dredge impact seems to identify a selection of species on Margherita bottoms. Indeed, *Astropecten spp.*, *L. depurator*, *B. brandaris* and *D. pugilator*, represent a set of species strongly associated to the impact of dredges, since these species can assume the role of scavengers [11, 12]. In fact, the mechanical disturbance and damage caused by the dredge on organisms makes them more vulnerable and they can be easily preyed upon by various opportunistic consumers [65]. In addition, some of these opportunistic predators are resistant to physical damage, such as *L. depurator*, *B. brandaris*, and *D. pugilator* [5, 6, 11]. In Barletta, the abundance of bivalves (*A. tubercolata*, *D. semistriatus*, *S. subtrnucata*) confirms the absence of relevant dredge impacts in the winter, since these species are generally sensitive to mechanical damage and the sand suspension from bottoms [6, 66].

A clear simplification of the assemblage structure was detected in the summer, with all investigated areas characterized by the intensity of fishing activities, apart from Varano. Overall, our results stress a general selection of more tolerant species, where the final structure of the assemblages is very similar in the areas within each fishing compartment. In particular, *D. pugilator*, *L. depurator*, *B. brandaris*, *Astropecten spp.* and *O. fusiformis* represent the characteristic species of summer assemblages. Their finding in the results of the multivariate analysis stresses the effect of dredge impacts on the assemblages. However, the IndVal analysis allows the detection of other species, which respond to environmental stress in the undredged area of Varano. In particular, *G. glycimeris* and *O. fusiformis* are the main characteristic species associated with the North Gargano area. The former is a filter feeder with a strong shell very resistant to the dredge impacts [5], while *O. fusiformis* benefits from high sediment resuspension in the dredge environment [66]. In addition, *M. stultorum* is the characteristic species in the Varano zone during the summer, indicating the absence of fishing impacts on the soft bottoms. This species is particularly sensitive to the dredge impacts and the sieving operations carried out to sort the catch [5, 11]. Another interesting outcome is the comparison between characteristic species in Margherita, where the assemblages seem to maintain a similar structure between the two seasons, despite the disappearance of some species in summer. Scavenger and opportunistic predators associated with low-medium impacts of dredges showed a similar importance in both seasons, while polychaetes *(N. hombergii*, *G. rouxii*) tend to disappear. This condition has been generally observed in all investigated areas and could be affected by dystrophic crisis with hypoxia events as reported on the long-term scale in the North Adriatic Sea [67].

The seasonal selection of the species shaped by fishing and environmental disturbances is reflected in the changes in the feeding traits pattern, where, in the summer, deposit feeders (bivalves, gastropods, echinoderms and polychaetes) and fish predators decreased, while filter suspension feeders (polychaetes), predators (echinoderms and gastropods), and opportunistic scavengers (decapod crustaceans) increased. This change in the feeding traits pattern is consistent with analysis conducted on benthic communities disturbed by hydraulic dredges [68], as well as with changes in benthic communities of shallow coastal lagoon in the Northern Adriatic Sea, where the temperature plays a key role in the selection process of more tolerant species [69]. In addition, the pattern seems to follow a spatial specificity during the winter, comparing the Varano area and those of Margherita and Barletta. Indeed, deposit and filter feeders seem to be the characteristic traits in the assemblages of Varano, while bivalve deposit feeders are mixed with opportunistic gastropods in the Barletta and Margherita areas. This could be explained by differences between the former and the latter areas in terms of natural and anthropogenic disturbances, among which could be considered the low fishing level detected in Margherita and Barletta (Table 1). In the summer, polychaetes filter feeders, represented by *O. fusiformis*, are dominant in the North Gargano area during the summer period, while other opportunistic/scavenger groups are associated to both fishing compartments. The increase in filter suspension feeders in the period of high fishing impacts could be attributed to the resuspension of sediments due to the impact of dredgers [12].

The main outcomes highlighted in this study indicate a complex interaction between fishing disturbance and environmental features, which shape the benthic communities along the Apulian coastline in relationship to intensity of fishing pressure, coastal anthropogenic pressures, and physical–chemical factors. The lack of environmental monitoring in situ during the sampling surveys has replaced by using remote sensing data obtained from Copernicus, which should be improved in term of the spatio-temporal scale. However, for the goal of this study focused on investigating changes in benthic assemblages along a large coastal area, the data adopted for the environmental characterization of the study area can be considered as satisfactory. From winter to summer, the general increase in temperature and net primary production, and the reduction of dissolved oxygen could be considered the main cause of stress for the assemblages in all areas. In addition, areas within the Barletta fishing compartment are also affected by higher salinity in the summer. These are considered structuring factors for the benthic community in eutrophic conditions [70], and their sudden variation between investigated seasons affects the selection of more tolerant species in the community. In addition, the net primary production seems to be a key factor in the difference between Varano and Lesina areas, where the former showed the lowest values in both seasons. Likely, a reduction in available energy for the food web occurred from west (Lesina) to east (Varano) along the coastline. This condition could represent another environmental factor shaping the differences between the benthic communities, likely influencing the size of organisms. Indeed, individuals of several species sampled in the Lesina stations resulted larger than those of Varano (personal communication). Although a similar observation was reported for the size distribution of coastal zooplankton communities in the North Gargano [71], further analysis should be carried out to understand the effects of changes in net primary production on the structure of the communities, as well as on the size distribution of investigated species. Furthermore, although direct observations are not available, the massive presence of tourists could increase the waste discharges in the area between Rodi and Varano, with effects on the sedimentation and turbidity of waters like those induced by dredges impacts.

In conclusion, information on the dredge impacts on the benthic habitats exploited by the clam fishery are very important in the perspective of attempting to implement a management strategy of the resource based on the EAF approach. These results represent a first step in ecological analysis aimed at updating the knowledge on the benthic assemblages impacted by hydraulic dredges, which were previously stuck in studies conducted in the 1990s in this area [23]. New evidence on the effects of fishing disturbances on the benthic organisms has been provided in this study, stressing the importance of evaluating the effect of other environmental variables on the macro-benthic communities impacted by dredges. Further studies should be addressed to better understand the ecological response of this community to multiple stressors, to support Sustainable Development Goal 14 “Life Below Water” [72], as well as assessments on the health status of marine ecosystems required by EU directives and fishery management plans.

### Supplementary Information

**Additional file 1.**

## Data Availability

All data generated or analysed during this study are included in this published article and its supplementary information files.
